# Optimization of Extraction and Enrichment of Steroidal Alkaloids from Bulbs of Cultivated *Fritillaria cirrhosa*

**DOI:** 10.1155/2014/258402

**Published:** 2014-04-01

**Authors:** Dongdong Wang, Shu Wang, Qingdan Du, Nanyi Wang, Simei Liu, Xiaoxia Wang, Jinghui Jiang

**Affiliations:** Department of Pharmacognosy, West China College of Pharmacy, Sichuan University, No. 17, Duan 3, Renmin Nan Road, Chengdu 610041, China

## Abstract

The bulbs of cultivated *Fritillaria cirrhosa* (BCFC) are used in China both for food and folk medicine due to its powerful biological activities. The aim of this study is to optimize the extraction and enrichment conditions of alkaloids from BCFC. Firstly, the orthogonal experimental design was used to optimize and evaluate four variables (ethanol concentration, solid-liquid ratio, extraction time, and temperature). Thereafter, resin adsorption was as a means to enrich alkaloids. Among 16 tested resins, H-103 resin presented higher adsorption capacity and desorption ratio. The equilibrium experimental data of the adsorption of total alkaloids, imperialine, and peimisine were well-fitted to the pseudo-first-order kinetics model, Langmuir and Freundlich isotherms models. Finally, in order to optimize the parameters for purifying alkaloids, dynamic adsorption and desorption tests were carried out. After one run treatment with H-103 resin, the contents of total alkaloids, imperialine, and peimisine in the product were 21.40-, 18.31-, and 22.88-fold increased with recovery yields of 94.43%, 90.57%, and 96.16%, respectively.

## 1. Introduction

*Fritillaria cirrhosa*, which belongs to the family Lilliaceae, is primarily distributed in the southwestern China. Bulbs of* Fritillaria cirrhosa* (BFC) have long been used both for food and folk medicine in many Asian countries [1]. The materials from wild collections can be harvested at least for five years [2]. In the past, the original plants of BFC were mainly obtained from their wild species and thus were unable to meet the demands of the industry [3]. In recent years, there is a significant breakthrough in artificial plantation technologies. The bulbs of cultivated* Fritillaria cirrhosa* (BCFC) have become the mainstream of BFC in market, which provide the adequate resources for further exploitation and utilization of BFC.

Studies on phytochemistry of BFC indicated that chemical components of BFC included steroidal alkaloids, saponins, terpenoids, glycosides, and many other compounds [4]. As revealed by modern studies, steroidal alkaloids were the major biological active compositions of BFC [5]. Pharmacological studies found that alkaloids from BFC exhibited remarkable antitussive, expectorant, antiasthmatic properties [6, 7], hypotensive effects [8], antibacterial activity, antitumor effects [9–11], anti-inflammatory effects [12, 13], and so on. However, the content of total alkaloids of BFC is quite low and varies wildly, approximately ranging from 0.02% to 0.3%, which is regarded as major quality constraint for its applications. To the best of our knowledge, there are few systematic publication focusing on optimization of the extraction and enrichment for large-scale production of alkaloids from BCFC; thus, there is an urgent need to optimize the extracting and enriching conditions.

Orthogonal array design (OAD) has been widely used to optimize the factors which influenced the extraction yield or extract profiles of bioactive components from natural materials [14]. It has been used to optimize experimental conditions with fewer numbers of experiments [15, 16]. In this study, OAD was also used to optimize the extraction conditions.

There are several conventional methods, such as polyamide chromatography, gel chromatography, and silica gel column, available for the enrichment of active constituents [14]. However, these methods have several disadvantages, including long time consuming poisonous residual solvents and low recoveries [17]. Recently, growing attention has been taken to enrich and purify targeted components from crude biological samples using macroporous resins for their convenience, low operating costs, low solvent consumption, high chemically stability, and easy regeneration [14, 17]. In this study, the absorption and desorption on macroporous resins were utilized for the enrichment of alkaloids from BCFC.

The present study focused on the optimization of extraction and enrichment of alkaloids from BCFC to develop a cost-effective method for large-scale extraction and enrichment of alkaloids. In extraction process, we tried to optimize four independent variables by using OAD. For enrichment of alkaloids, the optimum macroporous resin was screened and the major process parameters were determined. Meanwhile, the experimental isotherm data were analyzed using pseudo-first- and second-order models, the Langmuir and Freundlich equations. Finally, macroporous resin chromatography was used for the enrichment of alkaloids from BCFC.

## 2. Materials and Methods

### 2.1. Plant Materials and Reagents

BCFC were purchased from the dealers who cultivate the* Fritillaria cirrhosa* at Chengdu International Trade City Hehuachi Chinese Medicinal Herbal Market (Chengdu, Sichuan province, China) and identified by Professor Shu Wang (Department of Pharmacognosy, West China College of Pharmacy, Sichuan University, Chengdu, China). The sample (Wang 20120510) has been deposited in the pharmacognosy lab of West China College of Pharmacy, Sichuan University. Anhydrous ethanol, ammonia water, ethylenediamine, and chloroform were analytical grade obtained from Tianjing Kemiou Chemical Reagents Co. (Tianjing, China). Acetonitrile of chromatographic grade was purchased from Sigma Aldrich, Inc. (Steinheim, Germany). Imperialine and peimisine were isolated from BFC in our laboratory [12, 13]. The ultrapure water was prepared by a Milli-Q water system (Millipore, USA).

### 2.2. Selection of Extraction Parameters

The preprepared powder of BCFC (50 g) was soaked in 70 mL ammonia for different times (0.5 h, 1.0 h, 1.5 h, 2.0 h, 3.0 h, and 4.0 h) and then extracted under reflux with different concentrations of ethanol (50%, 60%, 70%, 80%, 90%, and 100%) with different ratios of liquid/solid (mL : g) (5 : 1, 10 : 1, 15 : 1, 20 : 1, 25 : 1, and 30 : 1) for different given times (60 min, 90 min, 120 min, 150 min, 180 min and 240 min), while the temperature of the water bath was set as 50°C, 60°C, 70°C, 80°C, 90°C, and 95°C, respectively, and kept steady (within ±2.0°C), for different extraction times (once, twice, thrice, and four times) [18].

### 2.3. Orthogonal Experimental Design of Extraction Procedure

Based on single-factor experimental results, four independent parameters (ethanol concentration (v/v, *C*), solid-liquid ratio (mL/g, *R*), extraction time (min, *t*), and temperature (°C, *T*) were confirmed as the major influencing factors; then a L_9_(3^4^) orthogonal experimental design was conducted to evaluate the effects of four independent variables on the extraction efficiency of total alkaloids, which was reflected by extraction yield of total alkaloids of BCFC. The range and levels of individual variables are shown in Table 1. The experiment design is shown in Table 2, along with experimental data. All experiments were run in twice.

### 2.4. Preparation of Sample Solution and Absorbents

The extract process was done according to optimal extraction conditions obtained in Section 2.3. The extracting solution was filtered, the filtrates were combined and evaporated using a vacuum rotary evaporator at 45°C and dried* in vacuo* for 72 h to yield ethanol extracts. The sample solution was prepared by dissolving the dried ethanol extracts in distilled water. The physical properties of the 16 kinds of adsorbents are summarized in Table 3. For prior use, the adsorbents needed to be soaked for 24 h with 100% ethanol and then washed with 1 mol/L HCl and NaOH solution successively and finally washed with distilled water to remove monomers and porogenic agents trapped inside the pores during the synthesis process [17].

### 2.5. Adsorption Resins Screening

#### 2.5.1. Adsorption Resins Screening

The optimum resin was screened by static adsorption and desorption tests. The adsorption tests on different resins were performed as follows: preweighed amounts of hydrated resins (equal to 0.5 g dry resin) and 100 mL sample solution (0.3271 mg/mL) were added into 250 mL flasks which were continually shaken for 24 h at 30°C with 120 rpm. The solutions after adsorption were separated from the resins and analyzed by Alpha-1900PC UV-Vis spectrophotometer and Shimadzu-10AT HPLC coupled to Sedex-75 evaporative light-scattering detector (HPLC-ELSD) according to the method described previously [19, 20]. The static desorption tests were conducted as follows: after reaching adsorption equilibrium, the resins were washed with 100 mL distilled water and then desorbed with 50 mL 80% ethanol solution. The flasks were continually shaken for 12 h at 30°C with 120 rpm. The adsorption and desorption properties of different resins for alkaloids including adsorption capacity, desorption capacity, and desorption ratio of each resin were quantified with the following equations [14, 21, 22]:

(1) $$\begin{array}{c} Q_{e}=\left(C_{0}-C_{e}\right)\times \frac{V_{i}}{W},\\ Q_{d}=\frac{C_{d}\times V_{d}}{W},\\ D=\frac{C_{d}\times V_{d}}{\left[\left(C_{0}-C_{e}\right)\times V_{i}\right]}\times 100, \end{array}$$

where *Q*_*e*_ is the adsorption capacity at adsorption equilibrium (mg/g dry resin); *Q*_*d*_ is the desorption capacity after desorption equilibrium (mg/g dry resin); *D* is the desorption ratio (%); *C*_0_, *C*_*e*_, and *C*_*d*_ are the initial, absorption equilibrium, and desorption concentrations of alkaloids in the solutions, respectively (mg/mL); *V*_*i*_ and *V*_*d*_ are the volume of the initial sample and desorption solution (mL), respectively; and *W* is the dry weight of tested resins (g).

To study the adsorption and desorption properties of the selected resin under different conditions, a series of sample solutions with pH values ranged from 5 to 10 for adsorption were tested, and a series of concentration of ethanol ranged from 10% to 100% for desorption were also investigated. All the solutions after adsorption and desorption at different conditions were analyzed by UV-Vis spectrophotometer [23].

#### 2.5.2. Adsorption Kinetics

Adsorption kinetics tests were conducted by mixing 0.5 g (dry weight) of hydrated H-103 resin with 100 mL of sample solutions (0.3078 mg/mL) in flasks. The concentrations of target compounds in the adsorption solution were monitored at different time intervals until equilibrium. Two most widely used adsorption models, pseudo-first-order and pseudo-second-order model, were used in determining the rate of the adsorption process to investigate the adsorption process of alkaloids of BCFC on H-103 resin [14, 21, 24].

The pseudo-first-order equation is given as follows:

(2) $$\begin{array}{c} \log\left(Q_{e}-Q_{t}\right)=\log\left(Q_{e}\right)-\left(\frac{k_{1}\times t}{2.303}\right), \end{array}$$

where *Q*_*e*_ and *Q*_*t*_ are the amounts of analyte (mg/g) adsorbed on the resin at equilibrium and at time *t*, respectively, and *k*_1_ is the rate constant of pseudo-first-order sorption.

The pseudo-second-order equation is expressed as follows:

(3) $$\begin{array}{c} \frac{t}{Q_{t}}=\frac{1}{k_{2}\times Q_{e}^{2}}+\frac{t}{Q_{e}}, \end{array}$$

where *k*_2_ is the rate constant of pseudo-second-order adsorption; *Q*_*e*_ and *Q*_*t*_ are the same as described above.

The linear intraparticle diffusion equation was further fitted using the adsorption kinetic data to determine whether intraparticle diffusion is the rate-limiting step [24]. The intraparticle diffusion equation is given as follows:

(4) $$\begin{array}{c} Q_{t}=k_{i}\times t^{1/2}+C, \end{array}$$

where *k*_*i*_ and *C* are the intraparticle diffusion rate constants; *Q*_*t*_ is the same as described above.

#### 2.5.3. Adsorption Isotherms

Afterwards, the tests for equilibrium adsorption isotherms on the selected resin were performed. 100 mL of sample solutions at different concentrations and preweighed amounts of hydrated resins (equal to 0.1 g dry resin) were added into 250 mL flasks and shaken for 24 h at different temperatures (20, 30, and 40°C). The effects of the initial concentration of sample solution and temperature on the adsorption characteristics were studied with the thermodynamic parameters of adsorption determined. Two standard theoretical models, well-known Langmuir model and Freundlich model, were frequently used to describe the adsorption behavior [21, 24].

The Langmuir isotherm equation is expressed as follows:

(5) $$\begin{array}{c} \frac{C_{e}}{Q_{e}}=\frac{C_{e}}{Q_{0}}+\frac{1}{K\times Q_{0}}, \end{array}$$

where *Q*_0_ is an empirical constant and *K* is the Langmuir adsorption equilibrium constant. *C*_*e*_ and *Q*_*e*_ are the same as described above.

The Freundlich isotherm equation is given as follows:

(6) $$\begin{array}{c} \ln Q_{e}=\ln k_{f}+\frac{1}{n}\times \ln C_{e}, \end{array}$$

where 1/*n* is an empirical constant and *k*_*f*_ is the Freundlich adsorption equilibrium constant [24]. *C*_*e*_ and *Q*_*e*_ are the same as described above.

The Gibbs free energy (Δ*G* ^0^) change indicates the degree of the spontaneity of the adsorption process [24]. The equation for Δ*G* ^0^ is defined as follows:

(7) ΔG0=−R×T×ln⁡k,ln⁡k=−ΔH0R×T+ΔS0R,

where *T* is temperature (*K*), Δ*H* ^0^ is the enthalpy of adsorption (kJ/mol), Δ*S* ^0^ is the entropy of adsorption (J/mol*·*K), and *k* is the equilibrium constant obtained from the best fitted model at 293, 303, or 313 K.

### 2.6. Dynamic Adsorption and Desorption Tests

#### 2.6.1. Effect of Concentration of Alkaloids in Sample Solution on Dynamic Adsorption

Dynamic adsorption tests were first carried out on glass columns (250 mm × 15 mm i.d.) wet-packed H-103 resin with the bed volume (BV) of 8 mL. The sample solution was loaded onto the macroporous resin columns at different concentrations of the loading sample solution (0.10, 0.20, and 0.40 mg/mL). The sample solution flowed through the resin column at a constant flow rate of 1.0 BV/h. The concentration of alkaloids in the aliquots of 1 mL effluents collected at 3.0 BV interval was monitored by UV-Vis spectrophotometer to get the dynamic breakthrough curve and select the most suitable concentration of the loading sample solution.

#### 2.6.2. Effect of Diameter to Height Ratio on Dynamic Adsorption

Besides, the effect of diameter-to-height ratio on breakthrough curve was examined in a similar way. The sample solution flowed through the columns (diameter-to-height ratio 1/3, 1/10, or 1/30) at a constant flow rate of 1.0 BV/h and the concentration of alkaloids in sample solution was approximate 0.20 mg/mL.

#### 2.6.3. Effect of Loading Flow Rate on Dynamic Adsorption

In adsorption process, in addition to concentration of the loading sample solution and diameter-to-height ratio, the effect of loading flow rate on breakthrough curve was also investigated. The sample solution was loaded onto the macroporous resin column (diameter-to-height ratio 1/10) at different flow rates of 2.0, 4.0, and 6.0 BV/h and the concentration of total alkaloids was approximately 0.20 mg/mL.

#### 2.6.4. Effect of Ethanol-Water Concentrations on Dynamic Desorption

After adsorption equilibrium under the optimum condition, the adsorbate-laden column was first washed with 8 BV of distilled water and 4 BV of 10% aqueous ethanol solution at a constant flow rate of 2 BV/h to elute impurities and then eluted with different concentrations of ethanol (70%, 80%, 90%, 95%) at the flow rate of 2 BV/h, respectively.

### 2.7. Laboratory Preparative-Scale Purification

The crude extracts from BFC (400.0 g) were dissolved in water. The sample solution (pH 7.0) was applied to a glass column (120.0 cm × 7.5 cm i.d.) containing 2.0 kg of wet H-103 macroporous resin with a bed volume (BV) of 2.5 L. Initially, distilled water was used to wash the elution solution until almost no color was noted, then 4 BV of 10% ethanol was used to remove the high polar components, and the adsorbent was finally rinsed with 6 BV of 90% ethanol to obtain the alkaloids-rich fraction. The flow rate of each gradient was set at about 2 BV/h, and the elution of 90% ethanol was collected, concentrated, and dried.

### 2.8. Statistical Analysis

The data were presented as means ± standard deviation (S. D.) and evaluated by one-way analysis of variance (ANOVA) using the Statistical Package for Social Sciences (SPSS) computer software program. *P* values <0.05 were regarded as significant.

## 3. Results and Discussion

### 3.1. Extraction Parameters

The extraction rate of constituents from crude resources was affected by many factors, such factors as extraction solvent, extraction time, temperature, and the ratio of liquid-solid, which were usually considered to have significant effect on compounds extraction rate [18, 25].

**Figure 1:**
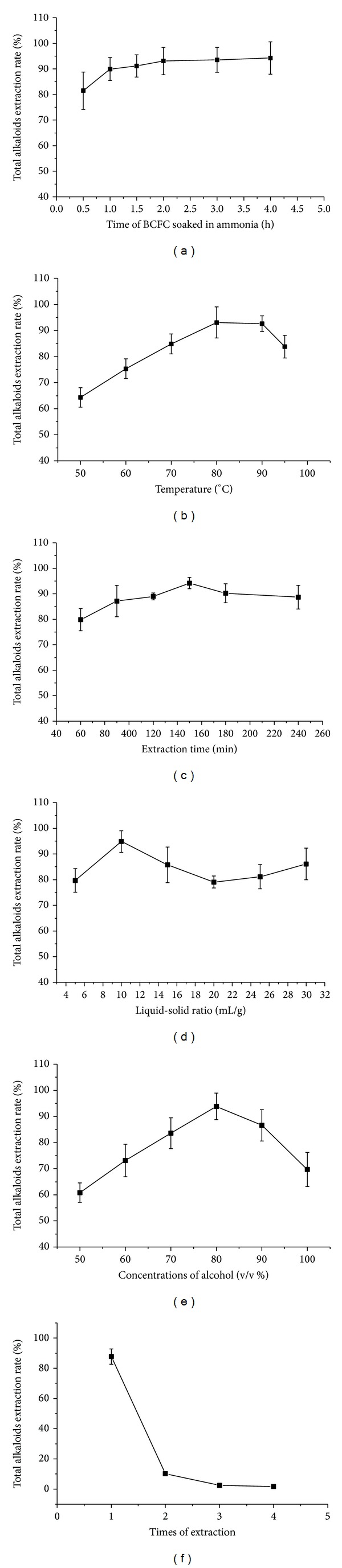
Effects of different parameters: the time of BCFC soaked in ammonia (a), temperature (b), extraction time (c), liquid-solid ratio (d), concentrations of alcohol (e), and times of extraction (f) on the total alkaloids extraction ratio.

All parameters were tested in a wider range prior to OAD optimization which help narrow down the ranges of the parameters tested. The effect of different parameters (the time of BCFC soaked in ammonia, concentrations of alcohol, extraction time, liquid-solid ratio, temperature, and times of extraction) on the extraction yield of alkaloids was shown in Figure 1. The results indicated that each selected parameter, except the time of BCFC soaked in ammonia and times of extraction, had a suitable level, at which the total alkaloids extraction rate reached the peak. It can be easily concluded from the corresponding figures that the extraction time 150 min, liquid-solid ratio 10 : 1, and concentrations of alcohol 80% were of suitable levels. So, concentrations of alcohol, liquid-solid ratio, extraction time, and temperature were selected as the four extraction parameters for the following OAD [18].

### 3.2. Optimization of Extraction Conditions

To verify whether the effect of individual factors on total alkaloids extraction efficiency is statistically significant, an ANOVA test was used to analyze the experimental data [25, 26]. The significance of each factor was evaluated by calculating the *R* value of extreme difference (Table 2) and the *F* value (Table 4). As seen in Tables 2 and 4, we concluded that the order of parameters influencing on extraction rate of total alkaloids was as follows: ethanol concentration > temperature > liquid-solid ratio > extraction time, and the four parameters were all statistically significant. Based on the analysis, the optimum conditions of extraction were therefore determined as follows: ethanol concentration (%, v/v) 90%, temperature 80°C, liquid-solid ratio 15 : 1, and extraction time 120 min. In order to validate the optimum conditions, a verification experiment was carried out. Under these conditions an extraction yield of total alkaloids from BCFC of 97.84% was obtained.

### 3.3. Screening of Optimum Resin

To obtain the most appropriate resins, absorption and desorption properties of sixteen macroporous resins towards total alkaloids, imperialine, and peimisine were assessed and the results were shown in Table 5. HPD-100, H-103, and HPD-722 resins exhibited notably higher adsorption capacity towards total alkaloids, imperialine, and peimisine than that of other resins. Moreover, the adsorption capacity of alkaloids on H-103 resin was the highest among the three resins. Besides, the desorption capacity and desorption ratio of HPD-722 resin for alkaloids were lower than that of HPD-100 and H-103 and desorption ratio of H-103 resin was close to that of HPD-100. In view of these results, H-103 resin was considered as the optimum one. H-103 resin exhibited better adsorption capacity and desorption ratio not only because of similar polarity with the nonpolar alkaloids from BCFC, but also because of its higher surface area which may correlate with the capability of the resin and the chemical features of the adsorbed substance [21].

### 3.4. Effects of pH on the Absorption Capacity and Ethanol Concentration on the Desorption Capacity

**Figure 2:**
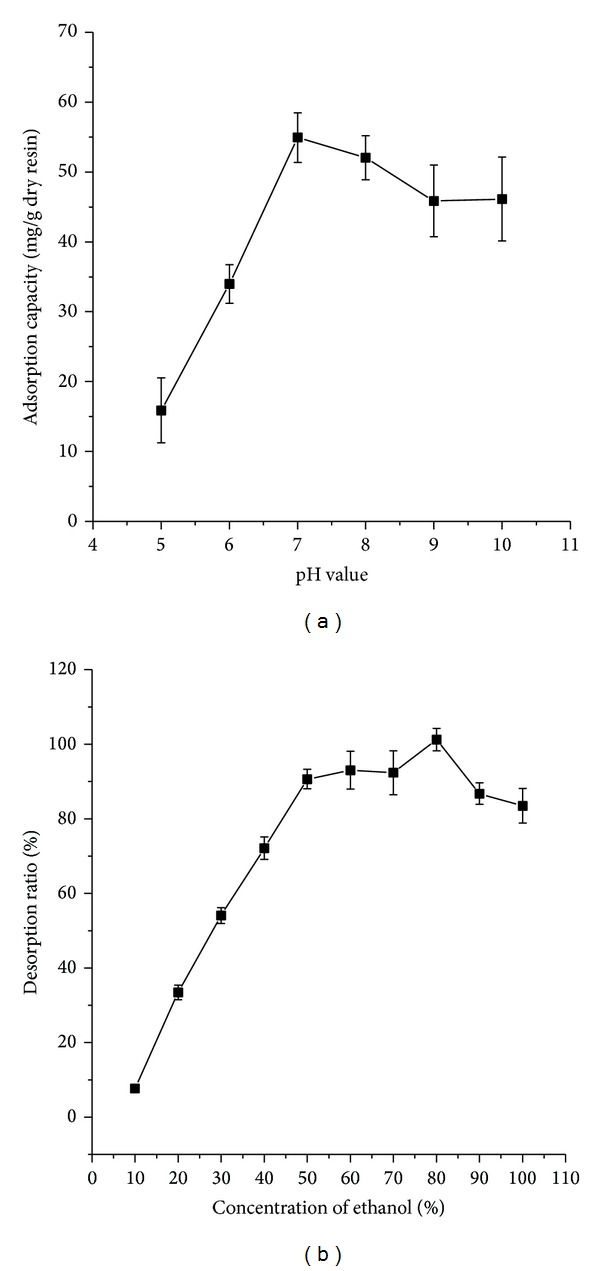
Effects of pH on the absorption capacity (a) and ethanol concentration on the desorption capacity (b) of total alkaloids.

The pH plays an important role in absorption processes because the ionization of solutes can be influenced by pH, thus affecting the absorption affinity between solutes and the adsorbents [14, 27]. The effect of pH of initial sample solution on the absorption capacity was shown in Figure 2(a). It shows that for total alkaloids the highest adsorption capacity appeared at the pH value of 7.0. The reason for the sharp decrease when the pH value was below 7.0 is that the alkaloids were ionized with decreasing of pH. In addition, increase of pH produces a lot of precipitation, which exerted negative influence on the absorption capacity. Based on these results, pH 7.0 of sample solutions was selected for absorption in the following tests.

Different concentrations of ethanol solutions were used to perform desorption experiments in order to choose proper desorption solution. As shown in Figure 2(b), the desorption ratios of alkaloids increased with the increase of ethanol concentration and reached their peak value at the concentration of 80%, then decreased with the increase of ethanol concentration [23].

### 3.5. Adsorption Kinetics

**Figure 3:**
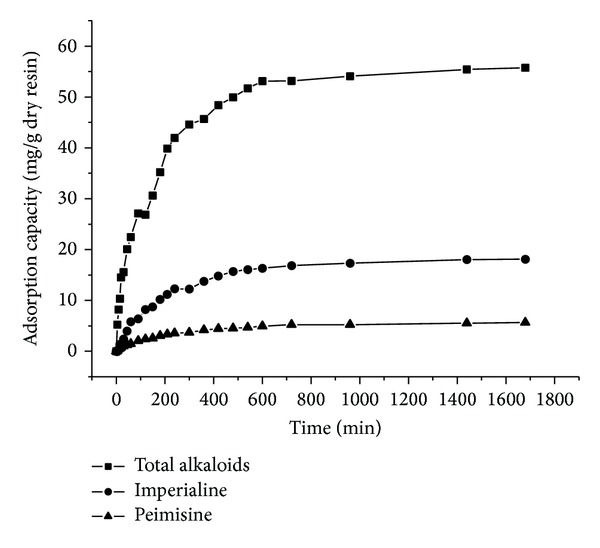
Adsorption kinetics curves of total alkaloids, imperialine, and peimisine on H-103 resin.

Adsorption kinetics of total alkaloids, imperialine, and peimisine on H-103 resin were investigated at 30°C and the adsorption kinetics curves were obtained. As shown in Figure 3, the adsorption of total alkaloids, imperialine, and peimisine increased with adsorption time before reaching equilibrium. In the first 240 min, the adsorption of total alkaloids rapidly increased and then increased slowly until adsorption equilibrium was reached at 600 min. The adsorption kinetics for imperialine and peimisine showed similar tendency with total alkaloids.

The kinetic parameters and correlation coefficients of the kinetic models were listed in Table 6. As shown in Table 6, the calculated *Q*_*e*_ of the pseudo-first-order equation values was closer to the experimental ones, and the correlation coefficients were almost higher than 0.95. Therefore, the first-order equation described the experimental data more accurately for total alkaloids, imperialine, and peimisine. On the other hand, the correlation coefficients for the pseudo-second-order kinetic model obtained were quite high, but the calculated *Q*_*e*_ values deviated significantly from the experimental ones. Thus, the adsorption observed did not fit the pseudo-second-order model.

The intraparticle diffusion model was used to evaluate the possibility of intraparticle diffusion. As shown in Table 6, based on the correlation coefficient of intraparticle diffusion model, *R* ^2^ indicated that pore diffusion was an important rate-limiting step.

### 3.6. Adsorption Isotherms

**Figure 4:**
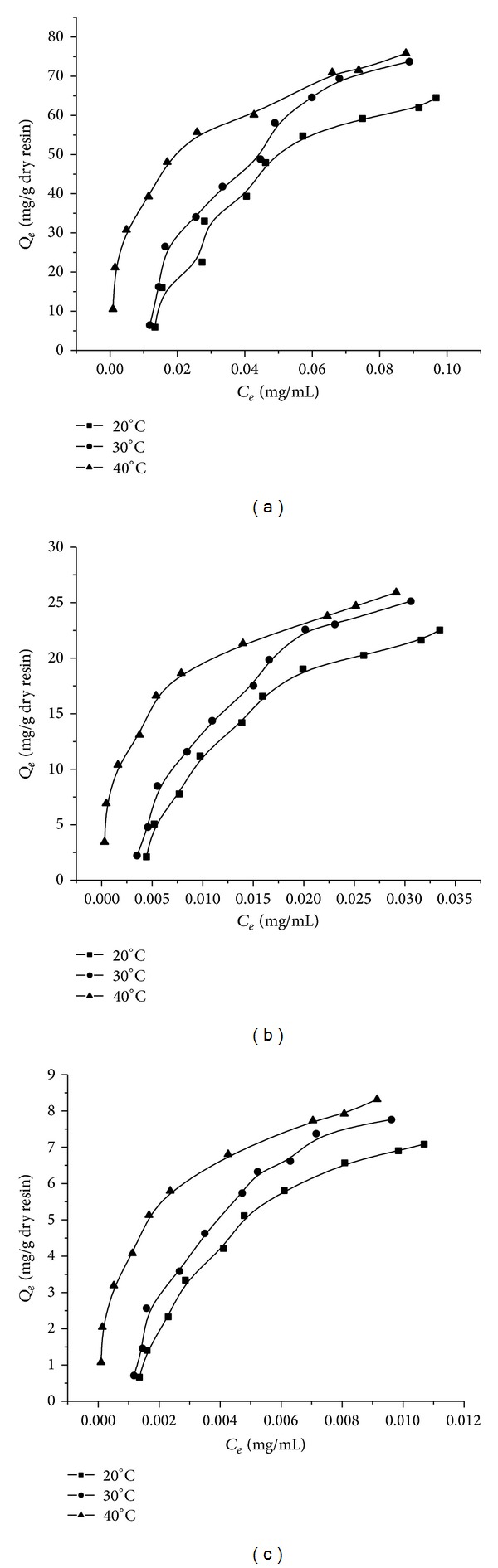
Adsorption isotherms of total alkaloids (a), imperialine (b), and peimisine (c) on H-103 resin at 20, 30, and 40°C.

The adsorption isotherms were shown in Figure 4. From the adsorption isotherm, the adsorption capacity increased with the initial concentration and reached the saturation plateau when the initial concentrations of total alkaloids, imperialine, and peimisine were 0.204 (Figure 4(a)), 0.074 (Figure 4(b)), and 0.024 mg/mL (Figure 4(c)), respectively. The adsorption isotherms for imperialine and peimisine showed similar tendency with that of total alkaloids.

The Langmuir and Freundlich equations are used to describe how solutes interact with the resins. The Langmuir model describes the monomolecular layer adsorption without mutual interaction between adsorbed molecules, while the Freundlich model is used to describe the adsorption behavior of monomolecular layer as well as that of the multimolecular layer [28]. As shown in Table 7, the correlation coefficients of both Langmuir and Freundlich equations for total alkaloids, imperialine, and peimisine were rather high. The experimental adsorption data were better fitted to Freundlich equation than Langmuir equation. In the Freundlich equation, values 1/*n* < 1.0 represent favorable adsorption conditions and values 1/*n* > 1.0 indicates difficulty in adsorption [24]. In Table 7, the 1/*n* values are between 0.1 and 1.0, which indicated that the adsorption of total alkaloids, imperialine, and peimisine on H-103 resin can take place easily. We also could see from Figure 4 that at a constant initial concentration, the adsorption capacities increased with temperature increase from 20 to 40°C, which indicated that the adsorption was an endothermic process.

The thermodynamic parameters of the adsorption process were listed in Table 8. Δ*G* ^0^ values were negative, indicating that the feasibility and spontaneity of the adsorption process. Its absolute values were less than the critical value (20 kJ/mol), showing that the adsorption process was physical [24, 29].

### 3.7. Dynamic Adsorption and Desorption on H-103 Resin

#### 3.7.1. Dynamic Leakage Curves on H-103 Resin

Adsorption presumably reached equilibrium when the leakage solution concentration was 5% or 10% of the initial concentration, which defined the leakage point [24, 30]. The breakthrough point in this study was set as 5% of the initial concentration.

**Figure 5:**
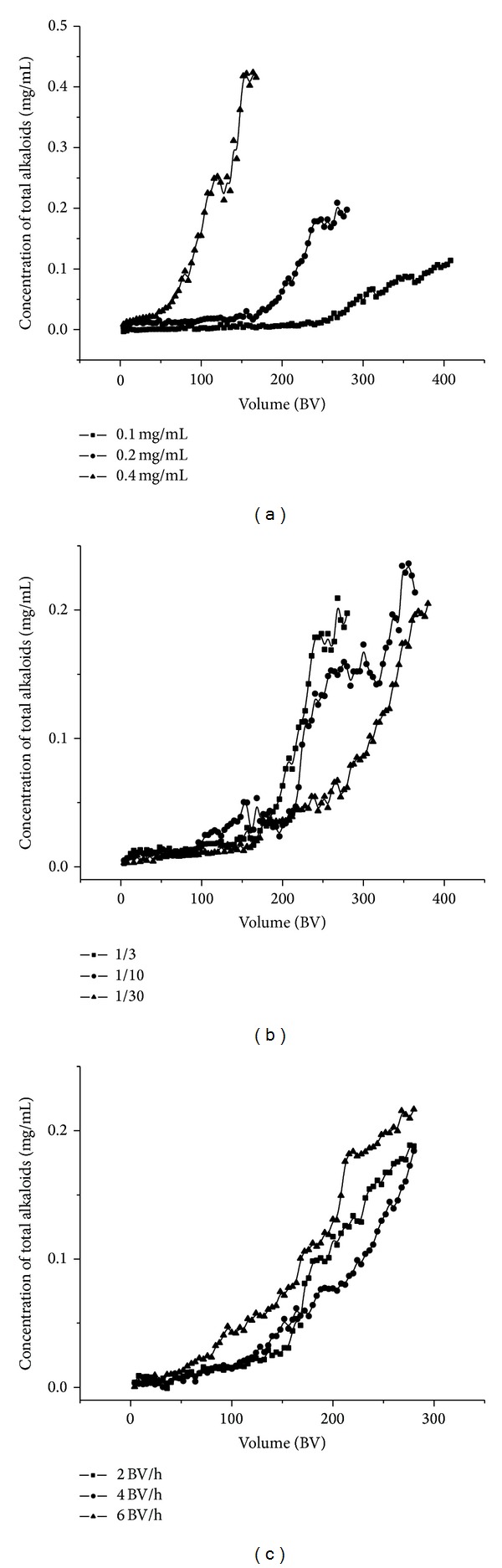
Dynamic breakthrough curves of total alkaloids on column packed with H-103 resin at different concentrations of sample (a), different diameter-to-height ratios (b), and at different sample flow rates (c).

As shown in Figure 5(a), the adsorption capacities at the leakage point were 118.24, 108.99, and 92.16 mg/8 mL resin at the concentrations of 0.10, 0.20, and 0.40 mg/mL, respectively. The adsorption capacities at the leakage point increased a little as the concentration decreased from 0.20 to 0.10 mg/mL, but increased significantly as the concentration decreased from 0.40 to 0.20 mg/mL. So the appropriate concentration of total alkaloids during the loading of the sample on the column was set as 0.20 mg/mL.

As shown in Figure 5(b), the adsorption capacities at the leakage point were 108.99, 143.76, and 162.95 mg/8 mL resin at the diameter to height ratio of 1/3, 1/10, and 1/30, respectively. The adsorption capacities increased significantly when the diameter-to-height ratio increased from 1/3 to 1/30. The significant increase in adsorption capacities was not observed when the diameter-to-height ratio increased from 1/10 to 1/30. Moreover, excessively more ratio of diameter-to-height was not conducive to large-scale application. So, a moderate diameter to height ratio of 1/10 was used in the following tests.

As shown in Figure 5(c), the adsorption capacities at the leakage point were 140.88, 110.81, and 83.42 mg/8 mL resin at the flow rate of 2, 4, and 6 BV/h, respectively. With the increase of the sample flow rate, the adsorption capacities at the leakage point reduced because the interaction time of alkaloids with active sites of the resin surface is decreased with the increase of sample flow rate [31]. The adsorption ratio was highest at a flow rate of 2.0 BV/h. Considering that the lower flow rate gave a long run time and was not conducive to industrial production, the flow rate for loading sample was maintained constantly at 4.0 BV/h. According to the above experiments, the corresponding breakthrough volumes of alkaloids on H-103 resin column of diameter-to-height ratio 1/10 were 88 BV at the concentrations of 0.2 mg/mL, at the flow rate of 4.0 BV/h (Figure 5(c)).

#### 3.7.2. Dynamic Desorption Curves on H-103 Resin

**Figure 6:**
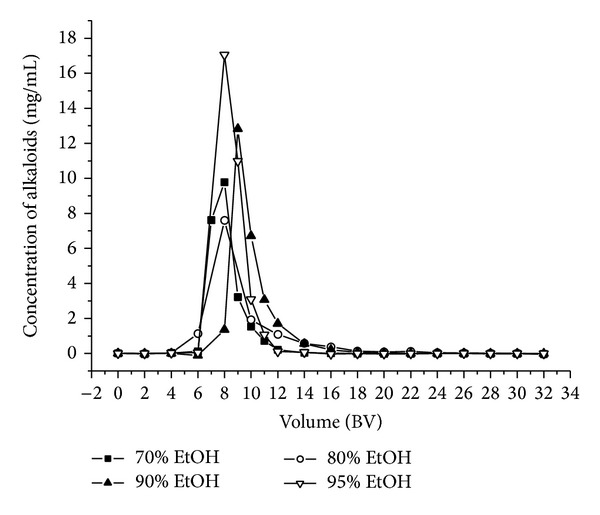
Dynamic desorption curves of total alkaloids on column packed with H-103 resin by different concentrations of ethanol solutions.

As shown in Section 3.4, at the 10% ethanol, alkaloids were hardly desorbed. The desorption ratios of total alkaloids reached their peak value at the concentration of 80% in static desorption. Therefore, we used different concentrations of ethanol (70%, 80%, 90%, and 95%) to establish the proper desorption condition on dynamic desorption. The results were shown in Figure 6. Samples after purification by H-103 resin were combined, concentrated in a rotary evaporator, and dried under vacuum. After dynamic desorption on H-103 resin column chromatography, the contents of total alkaloids in the products which were eluted by 70%, 80%, 90%, and 95% ethanol were 486.14, 550.89, 638.48, and 643.20 mg/g, respectively, which were 17.23-, 19.53-, 22.63-, and 22.80-fold increased. Their recoveries which were eluted by 70%, 80%, 90%, and 95% ethanol were 95.75%, 96.64%, 95.87%, and 95.46%, respectively. Considering efficiency and economy of the process, 90% ethanol was selected as the desorption solution in the dynamic desorption experiment. Thus, the optimal enrichment conditions for alkaloids on H-103 resin were as follows: for adsorption: initial concentrations of total alkaloids in sample solution: 0.20 mg/mL, diameter-to-height ratio of resin column: 1/10, loading flow rate: 2 BV/h, feed volume: 88 BV, and pH 7.0; temperature: 40°C and for desorption: a successive gradient elution: 8 BV distilled water, 4 BV 10% ethanol, 6 BV 90% ethanol, and flow rate: 2 BV/h.

### 3.8. Laboratory Preparative-Scale Purification

**Figure 7:**
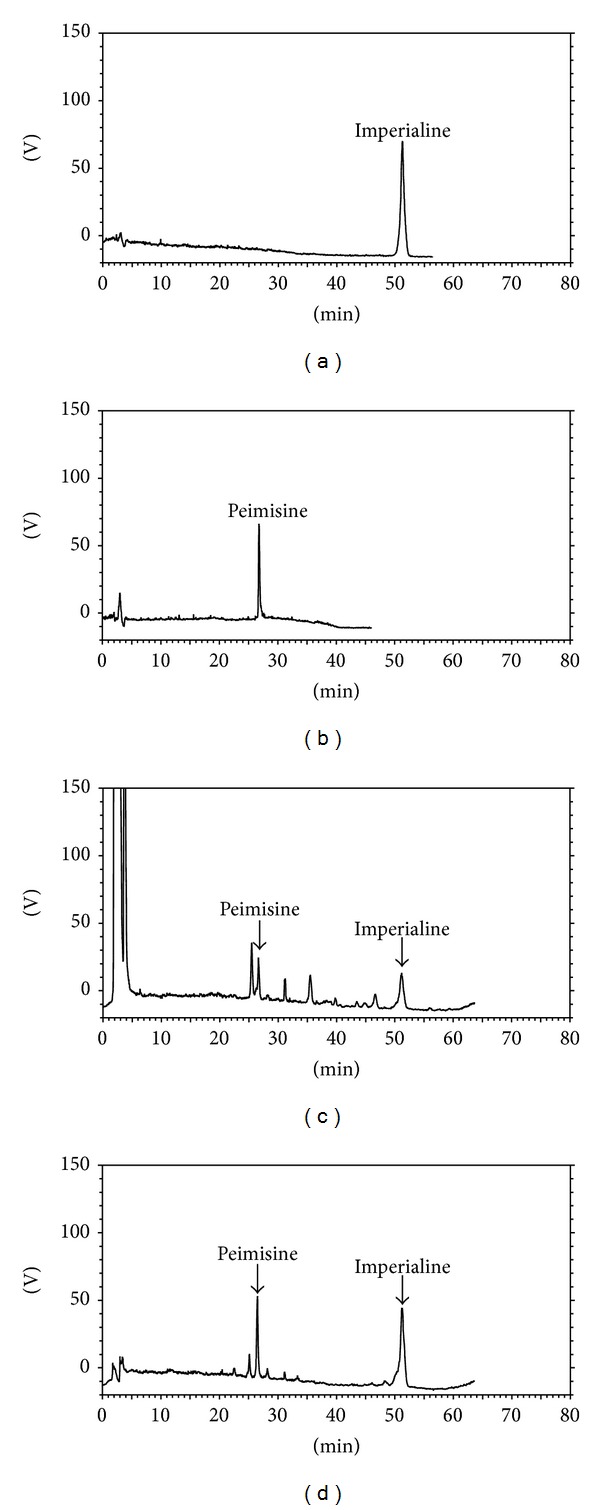
HPLC chromatograms of imperialine (a), peimisine (b), sample before treatment (c), and fraction eluted by 90% ethanol (d).

All methods used for purifying alkaloids from BCFC were investigated in a small-scale preparation. This was followed by a large-scale preparation of pure alkaloids using the above determined conditions. The elution of 6 BV 90% ethanol gave the alkaloids-rich fraction, in which the contents of total alkaloids, imperialine, and peimisine were 21.40-, 18.31-, and 22.88-fold increased with recovery yields of 94.43%, 90.57%, and 96.16%, respectively. The HPLC chromatograms of the experimental samples before and after purification with H-103 resin were shown in Figure 7.

## 4. Conclusions

The present study established an effective extraction and enrichment procedure for alkaloids from BCFC. On the one hand, the extraction of alkaloids from BCFC was investigated with a four-variable, three-level experiment orthogonal experimental design in enhancing the alkaloids extraction yield. On the other hand, this study reports resin adsorption as a means to enrich alkaloids from BCFC extracts was successfully developed. Among 16 tested resins, H-103 resin presented higher adsorption capacity and desorption ratio. The equilibrium experimental data of the adsorption of total alkaloids, imperialine, and peimisine on H-103 resin at different temperatures were well-fitted to the pseudo-first-order kinetics model, Langmuir and Freundlich isotherms models. The processes of dynamic adsorption and desorption were conducted to ensure the optimal purification parameters of the H-103 resin. To the best of our knowledge, this is the first systematic publication focusing on optimization of the extraction and enrichment for large-scale production of total alkaloids from BCFC. In conclusion, the developed methodology can also be referenced for the extraction and purification of other active compounds from herbal materials and large-scale manufacture of alkaloids of BCFC in food and pharmaceutical industry.

## Figures and Tables

**Table 1:** Factors and levels of orthogonal experimental design.

Levels	Ethanol concentration (*C*, v/v%)	Solid-liquid ratio (*R*, mL/g)	Extraction time (*t*, min)	Temperature (*T*, °C)
1	80	5 : 1	60	70
2	90	10 : 1	90	80
3	100	15 : 1	120	90

**Table 2:** Orthogonal experimental design for extraction of total alkaloids from BCFC.

Test no.	*t* (min)	*C* (v/v%)	*T* (°C)	*R* (mL/g)	*Y* (%)
1	1	1	1	1	81.42 ± 2.78
2	1	2	2	2	96.63 ± 0.98
3	1	3	3	3	63.81 ± 4.65
4	2	1	2	3	107.41 ± 3.28
5	2	2	3	1	80.95 ± 6.62
6	2	3	1	2	81.73 ± 3.78
7	3	1	3	2	91.99 ± 4.80
8	3	2	1	3	106.73 ± 3.64
9	3	3	2	1	78.84 ± 2.15
*k*_1_	80.62	93.61	89.96	80.41	
*k*_2_	90.03	94.77	94.29	90.11	
*k*_3_	92.52	74.80	78.92	92.65	
*R*	11.90	19.98	15.38	12.25	

*Y*: mean value of extraction yield of total alkaloids; *R* is the difference between the maximum value and the minimum value of *k*_*i*_ of any columns. The reported values are expressed as mean ± S.D. (*n* = 2).

**Table 3:** Physical properties of the macroporous resins.

Name	Polarity	Particle diameter (mm)	Surface area (m^2^/g)	Average pore diameter (nm)
HPD-100	Nonpolar	0.3–1.20	650–700	8.5–9.0
HPD-200A	Nonpolar	0.3–1.25	700–750	8.5–9.0
HPD-300	Nonpolar	0.3–1.20	800–870	5.0–5.5
HPD-700	Nonpolar	0.3–1.20	650–700	8.5–9.0
D-101	Nonpolar	0.2–0.60	400–600	10.0–12.0
D-3520	Nonpolar	0.3–1.25	480–520	8.5–9.0
D-4006	Nonpolar	0.3–1.25	400–440	6.5–7.5
H-103	Nonpolar	0.3–1.25	900–1100	8.4–9.4
HPD-450A	Middle-polar	0.3–1.25	500–550	9.0–10.0
HPD-750	Middle-polar	0.3–1.20	650–700	8.5–9.0
ADS-17	Middle-polar	0.3–1.25	90–150	20.0–30.0
DM-130	Middle-polar	0.3–1.25	500–550	9.0–10.0
HPD-722	Weak-polar	0.3–1.25	485–530	13.0–14.0
AB-8	Weak-polar	0.3–1.25	480–520	13.0–14.0
HPD-600	Polar	0.3–1.20	550–600	8.0
HPD-826	Polar	0.3–1.25	500–600	9.0–10.0

**Table 4:** Variance analysis of orthogonal experimental data.

Source of variance	Sum of square	Degree of freedom	Mean square	*F* value
Extraction time	472.780	2	236.390	15.176**
Ethanol concentration	1508.467	2	754.233	48.422***
Temperature	754.461	2	377.231	24.218***
Solid-liquid ratio	501.241	2	250.620	16.090**
Error	140.187	9	15.576	

Total	141896.623	18		

*F* critical value (95%) = 4.25; **P < 0.01; ***P < 0.001.

**Table 5:** Adsorption capacity, desorption capacity, and desorption ratio of different resins for total alkaloids, imperialine, and peimisine at 30°C.

Resin	Adsorption capacity (mg/g dry resin)			Desorption capacity (mg/g dry resin)			Desorption ratio (%)		
	Total alkaloids	Imperialine	Peimisine	Total alkaloids	Imperialine	Peimisine	Total alkaloids	Imperialine	Peimisine
HPD-100	57.76 ± 3.01	20.58 ± 3.56	5.71 ± 0.76	53.01 ± 5.23	18.79 ± 2.13	5.09 ± 0.29	91.67 ± 4.28	91.80 ± 5.54	89.46 ± 6.87
HPD-200A	46.45 ± 2.53	16.75 ± 2.33	5.05 ± 0.32	43.94 ± 0.54	15.73 ± 1.97	4.56 ± 0.07	94.71 ± 4.01	94.00 ± 1.28	90.42 ± 4.39
HPD-300	56.09 ± 5.24	19.83 ± 2.46	5.20 ± 0.44	44.92 ± 1.90	16.46 ± 2.86	4.20 ± 0.64	80.27 ± 4.12	82.72 ± 4.18	80.52 ± 5.53
HPD-700	54.85 ± 0.75	19.33 ± 1.05	5.54 ± 0.40	47.77 ± 1.54	16.49 ± 1.71	4.69 ± 0.03	87.10 ± 3.99	85.17 ± 4.23	84.92 ± 5.58
D-101	50.62 ± 1.84	18.01 ± 1.44	4.62 ± 0.48	39.67 ± 2.86	13.96 ± 0.70	3.76 ± 0.17	78.52 ± 8.52	77.59 ± 2.34	81.46 ± 4.76
D-3520	51.16 ± 4.37	18.45 ± 2.00	4.92 ± 0.62	42.96 ± 7.57	14.74 ± 2.53	4.09 ± 0.30	83.66 ± 7.65	79.59 ± 5.08	83.51 ± 4.40
D-4006	54.55 ± 1.94	19.37 ± 2.54	5.60 ± 0.59	48.31 ± 3.15	16.95 ± 0.85	4.94 ± 0.99	88.72 ± 8.93	87.98 ± 7.11	87.69 ± 8.42
H-103	59.40 ± 5.17	21.07 ± 2.78	5.96 ± 0.35	53.54 ± 2.03	19.06 ± 1.71	5.30 ± 0.49	90.33 ± 4.45	90.71 ± 3.86	88.89 ± 3.10
HPD-450A	46.63 ± 1.67	16.82 ± 0.81	4.62 ± 0.37	39.64 ± 1.92	14.16 ± 0.81	3.81 ± 0.64	84.99 ± 1.06	84.41 ± 8.86	82.18 ± 7.32
HPD-750	52.23 ± 2.92	18.74 ± 3.17	5.31 ± 0.88	45.43 ± 6.22	15.95 ± 1.73	4.43 ± 0.43	86.78 ± 7.06	85.55 ± 5.25	83.89 ± 5.77
ADS-17	49.38 ± 0.71	17.06 ± 1.32	4.94 ± 0.37	42.29 ± 1.95	14.22 ± 0.18	4.08 ± 0.02	85.63 ± 2.72	83.64 ± 7.51	82.92 ± 6.70
DM-130	11.05 ± 1.10	3.99 ± 1.39	1.56 ± 0.24	13.13 ± 2.04	4.79 ± 1.93	1.83 ± 0.41	118.43 ± 6.63	116.01 ± 7.13	109.28 ± 8.43
HPD-722	57.64 ± 2.61	20.39 ± 1.51	5.74 ± 0.23	48.65 ± 1.36	15.95 ± 2.56	4.44 ± 0.16	84.54 ± 6.18	77.97 ± 6.78	77.34 ± 5.97
AB-8	56.33 ± 1.25	19.51 ± 1.58	6.16 ± 0.57	52.10 ± 5.46	17.44 ± 3.06	5.48 ± 0.94	92.41 ± 7.64	89.01 ± 8.47	88.71 ± 6.93
HPD-600	43.26 ± 5.71	15.30 ± 2.48	4.46 ± 0.51	37.90 ± 3.71	13.13 ± 2.82	3.79 ± 0.67	87.82 ± 3.02	85.42 ± 4.58	84.68 ± 5.22
HPD-826	41.83 ± 5.53	14.59 ± 2.70	4.87 ± 0.81	42.12 ± 8.56	13.89 ± 2.91	4.51 ± 0.95	100.20 ± 7.22	95.04 ± 2.32	92.24 ± 4.29

**Table 6:** Kinetics parameters and correlation coefficients for the adsorption of total alkaloids, imperialine, and peimisine on H-103 resin.

Compound	*Q*_*e*_, exp^a^ (mg/g)	Pseudo-first-order			Pseudo-second-order			Intraparticle diffusion		
		*Q*_*e*_, cal^b^ (mg/g)	*k*_1_ (min^−1^)	*R* ^2^	*Q*_*e*_, cal^b^ (mg/g)	*k*_2_ (g/mg·min)	*R* ^2^	*C* (mg/g)	*k*_*i*_ (mg/g·min^−1/2^)	*R* ^2^
Total alkaloids	55.745	49.627	3.661 × 10^−3^	0.971	1.057	2.085 × 10^−3^	0.997	10.568	1.492	0.846
Imperialine	18.106	16.482	3.651 × 10^−3^	0.990	1.178	0.287 × 10^−3^	0.958	1.271	0.540	0.878
Peimisine	5.656	4.608	2.730 × 10^−3^	0.967	1.092	1.172 × 10^−3^	0.990	0.457	0.161	0.904

^a^Exp is expressed as experimental value; ^b^Cal is expressed as calculated value.

**Table 7:** Langmuir and Freundlich adsorption isotherm parameters of total alkaloids, imperialine and peimisine on H-103 resin at different temperatures.

Compound	Temperature (°C)	Langmuir equation	*Q*_0_ (mg/g)	*K*	*R* ^2^	Freundlich equation	*k*_*f*_	1/*n*	*R* ^2^
Total alkaloids	20	*C*_*e*_/*Q*_*e*_ = 0.00841*C*_*e*_ + 0.00065	114.340	13.923	0.952	ln⁡*Q*_*e*_ = 6.062 + 0.764 × ln⁡*C*_*e*_	429.196	0.764	0.944
	30	*C*_*e*_/*Q*_*e*_ = 0.00823*C*_*e*_ + 0.00047	121.557	17.385	0.954	ln⁡*Q*_*e*_ = 6.010 + 0.670 × ln⁡*C*_*e*_	407.666	0.670	0.993
	40	*C*_*e*_/*Q*_*e*_ = 0.01246*C*_*e*_ + 0.00011	80.267	111.555	0.990	ln⁡*Q*_*e*_ = 5.324 + 0.378 × ln⁡*C*_*e*_	205.232	0.378	0.953

Imperialine	20	*C*_*e*_/*Q*_*e*_ = 0.02505*C*_*e*_ + 0.00063	39.912	39.317	0.939	ln⁡*Q*_*e*_ = 5.756 + 0.742 × ln⁡*C*_*e*_	315.965	0.742	0.978
	30	*C*_*e*_/*Q*_*e*_ = 0.02178*C*_*e*_ + 0.00051	45.901	38.903	0.976	ln⁡*Q*_*e*_ = 6.048 + 0.762 × ln⁡*C*_*e*_	423.471	0.762	0.989
	40	*C*_*e*_/*Q*_*e*_ = 0.03637*C*_*e*_ + 0.00011	27.493	325.897	0.992	ln⁡*Q*_*e*_ = 4.710 + 0.388 × ln⁡*C*_*e*_	111.089	0.388	0.946

Peimisine	20	*C*_*e*_/*Q*_*e*_ = 0.07734*C*_*e*_ + 0.00014	12.930	121.359	0.976	ln⁡*Q*_*e*_ = 5.537 + 0.756 × ln⁡*C*_*e*_	253.900	0.756	0.984
	30	*C*_*e*_/*Q*_*e*_ = 0.07734*C*_*e*_ + 0.00014	16.454	108.524	0.926	ln⁡*Q*_*e*_ = 5.968 + 0.799 × ln⁡*C*_*e*_	390.781	0.799	0.986
	40	*C*_*e*_/*Q*_*e*_ = 0.11229*C*_*e*_ + 0.00011	8.905	1006.130	0.992	ln⁡*Q*_*e*_ = 4.080 + 0.399 × ln⁡*C*_*e*_	59.119	0.399	0.955

**Table 8:** The thermodynamic parameters of adsorption of total alkaloids, imperialine, and peimisine on H-103 resin at different temperatures.

Compound	*k* (L/mg)			Δ*G* ^0^ (kJ/mol)		
	293 K	303 K	313 K	293 K	303 K	313 K
Total alkaloids	13.923	39.317	121.359	−6.415	−7.194	−12.268
Imperialine	17.385	38.903	108.524	−8.944	−9.223	−15.058
Peimisine	111.555	325.897	1006.130	−11.690	−11.807	−17.992

## References

[1] Li XW, Song JY, Wei JH, Hu ZG, Xie CX, Luo GA (2012). Natural Fostering in *Fritillaria cirrhosa*: integrating herbal medicine production with biodiversity conservation. *Acta Pharmaceutica Sinica B*.

[2] Li X, Dai Y, Chen S (2009). Growth and physiological characteristics of *Fritillaria cirrhosa* in response to high irradiance and shade in age-related growth phases. *Environmental and Experimental Botany*.

[3] Jin L, Ding L, Luo GH, Chen Z, Ma YG (2009). Effects of different concentration GA3 on germination rate and esterase of *Fritillaria cirrhosa* seeds. *Seed*.

[4] Hao DC, Gu XJ, Xiao PG, Peng Y (2013). Phytochemical and biological research of *Fritillaria* medicinal resources. *Chinese Journal of Natural Medicine*.

[5] Li S, Liu J, Gong X, Yang XL, Zhu YG, Cheng Z (2013). Characterizing the major morphological traits and chemical compositions in the bulbs of widely cultivated *Fritillaria* species in China. *Biochemical Systematics and Ecology*.

[6] Chen MH Studies on *Fritillaria cirrhosa* D. Don and its effects of antitussive and antiasthma.

[7] Yan XY Effects of ethanol extract of three kinds of Bulb *Fritillariae cirrhosae* on guinea pigs with allergic asthma.

[8] Kang DG, Sohn EJ, Lee YM (2004). Effects of bulbus *Fritillaria* water extract on blood pressure and renal functions in the L-NAME-induced hypertensive rats. *Journal of Ethnopharmacology*.

[9] Wang DD, Wang S, Feng Y (2014). Antitumor effects of Bulbus *Fritillariae cirrhosae* on Lewis lung carcinoma cells *in vitro* and *in vivo*. *Industrial Crops and Products*.

[10] Wang DD, Feng Y, Li Z (2013). *In vitro* and *in vivo* antitumor activity of Bulbus *Fritillariae cirrhosae* and preliminary investigation of its mechanism. *Nutrition and Cancer*.

[11] Li Y, Xu C, Zhang Q, Liu JY, Tan RX (2005). *In vitro* anti-Helicobacter pylori action of 30 Chinese herbal medicines used to treat ulcer diseases. *Journal of Ethnopharmacology*.

[12] Wang D, Zhu J, Wang S (2011). Antitussive, expectorant and anti-inflammatory alkaloids from Bulbus *Fritillariae cirrhosae*. *Fitoterapia*.

[13] Wang D, Wang S, Chen X (2012). Antitussive, expectorant and anti-inflammatory activities of four alkaloids isolated from Bulbus of *Fritillaria wabuensis*. *Journal of Ethnopharmacology*.

[14] Wang CY, Shi LL, Fan LT (2013). Optimization of extraction and enrichment of phenolics from pomegranate (*Punica granatum* L.) leaves. *Industrial Crops and Products*.

[15] Guo L, Cho SY, Kang SS, Lee S-H, Baek H-Y, Kim YS (2007). Orthogonal array design for optimizing extraction efficiency of active constituents from Jakyak-Gamcho Decoction, the complex formula of herbal medicines, Paeoniae Radix and Glycyrrhizae Radix. *Journal of Ethnopharmacology*.

[16] Wang S-C, Liao H-J, Lee W-C, Huang C-M, Tsai T-H (2008). Using orthogonal array to obtain gradient liquid chromatography conditions of enhanced peak intensity to determine geniposide and genipin with electrospray tandem mass spectrometry. *Journal of Chromatography A*.

[17] Liu CQ, Zhang P, Liu L (2013). Isolation of *α*-arbutin from *Xanthomonas* CGMCC 1243 fermentation broth by macroporous resin adsorption chromatography. *Journal of Chromatography B*.

[18] Lu C-L, Li Y-M, Fu G-Q (2011). Extraction optimisation of daphnoretin from root bark of *Wikstroemia indica* (L.) C.A. and its anti-tumour activity tests. *Food Chemistry*.

[19] National Commission of Chinese Pharmacopoeia (2008). Pharmacopoeia of Peoples Republic of China. *China Medical Science Press*.

[20] Wang C, Wang S, Ma J (2010). HPLC fingerprint of Bullbus *Fritillariae cirrhosae*. *West China Journal of Pharmaceutics*.

[21] Wei Z, Zu Y, Fu Y (2011). Resin adsorption as a means to enrich rare stilbenes and coumarin from pigeon pea leaves extracts. *Chemical Engineering Journal*.

[22] Zhang Z-F, Liu Y, Luo P, Zhang H (2009). Separation and purification of two flavone glucuronides from *Erigeron multiradiatus* (Lindl.) Benth with macroporous resins. *Journal of Biomedicine and Biotechnology*.

[23] Zhao Z, Dong L, Wu Y, Lin F (2011). Preliminary separation and purification of rutin and quercetin from *Euonymus alatus* (Thunb.) Siebold extracts by macroporous resins. *Food and Bioproducts Processing*.

[24] Mi JN, Zhang M, Ren GX, Zhang HY, Wang YR, Hu P (2012). Enriched separation of protopanaxatriol ginsenosides, malonyl ginsenosides and protopanaxadiol ginsenosides from *Panax ginseng* using macroporous resins. *Journal of Food Engineering*.

[25] Martin MT, Yu Y, Cui ZQ, Zhang Y (2012). Optimization and orthogonal design of an ultrasonic-assisted aqueous extraction process for extracting chlorogenic acid from dry tobacco leaves. *Chinese Journal of Natural Medicine*.

[26] Zhou SK, Bi TN, Xu YF, Zhang RL, Yang MJ (2013). Extraction optimization of carbohydrate compound from Huangqi using orthogonal design. *International Journal of Biological Macromolecules*.

[27] Fu Y, Zu Y, Liu W (2007). Preparative separation of vitexin and isovitexin from pigeonpea extracts with macroporous resins. *Journal of Chromatography A*.

[28] Wang J, Wu FA, Zhao H, Liu L, Wu QS (2008). Isolation of flavonoids from mulberry (*Morus alba* L.) leaves with macroporous resins. *African Journal of Biotechnology*.

[29] Bell JP, Tsezos M (1987). Removal of hazardous organic pollutants by biomass adsorption. *Journal of the Water Pollution Control Federation*.

[30] Fu Y, Zu Y, Li S (2008). Separation of 7-xylosyl-10-deacetyl paclitaxel and 10-deacetylbaccatin III from the remainder extracts free of paclitaxel using macroporous resins. *Journal of Chromatography A*.

[31] Liu W, Zhang S, Zu Y-G (2010). Preliminary enrichment and separation of genistein and apigenin from extracts of pigeon pea roots by macroporous resins. *Bioresource Technology*.

